# Supplementary material for: Photochemistry and Photophysics of Cholesta-5,7,9(11)-trien-3β-ol in Ethanol
Source: Molecules. 2023 May 14;28(10):4086. doi: 10.3390/molecules28104086 (PMC10222123; doi:10.3390/molecules28104086)
Supplement: Supplementary file 1 [file molecules-28-04086-s001.zip › molecules-2386563-supplementary.pdf]

Photochemistry and Photophysics of Cholesta-5,7,9(11)-trien-3β-ol in Ethanol

Jack Saltiel *, Sumesh B. Krishnan, Shipra Gupta, Anjan Chakraborty,
Edwin F. Hilinski and Xinsong Lin

Department of Chemistry and Biochemistry, Florida State University,
Tallahassee, FL 32306-4390, USA

Supporting Information: Figure S1, ^{1}H NMR spectrum of the final product mixture from the irradiation of HOCTL in Ar outgassed ethanol. Figure S2, the UV spectrum of HOE_2. Figures S3-S6, ^{1}H NMR spectra of HOD_1, HOD_2, HOE_1 and HOE_2, respectively. Figure S7, COSY ^{1}H NMR spectrum of HOE_1. Figure S8, NOESY ^{1}H NMR spectrum of HOE_1. Figure S9, Expanded region of Figure S7 showing the correlation between the vinyl H at C_{11} and the H-6 proton. Figure S10, NOESY ^{1}H NMR spectrum of HOE_1 in CD_3OD. Figure S11, ^{1}H NMR spectrum of $HORP_2$ in CD_3Cl. Figure S12, Time evolution of UV spectra from the 313 nm irradiation of CTL in ethanol. Figure S13, Combination coefficient plot for the spectra in Figure S12. X-ray structures: Tables S1-S7 of X-ray data consisting of crystallographic parameters, positional parameters, bond distances, bond angles, and torsional angles for HOCTL. Tables S8-S14 and Tables S15-S21 for HOCTLP and $HORP_2$, respectively. Table S22, Hydrogen Atom Coordinates (Å × 10^4) and Isotropic Displacement Parameters (Å2 × 10^3) for $HORP_2$. Reference [1] in Supporting Information is cited as reference [20] in the manuscript.

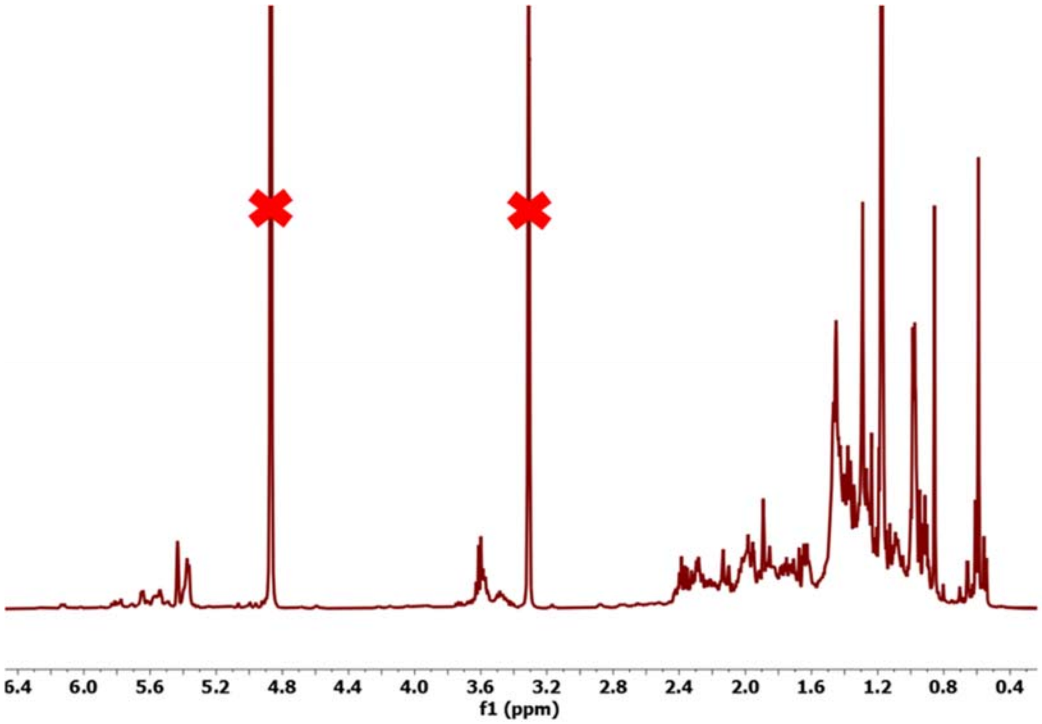

Figure S1. ^{1}H NMR spectrum in CD_3OD of photoproduct mixture from the irradiation of HOCTL in Ar outgassed ethanol. The crossed out peaks belong to partially protonated solvent.

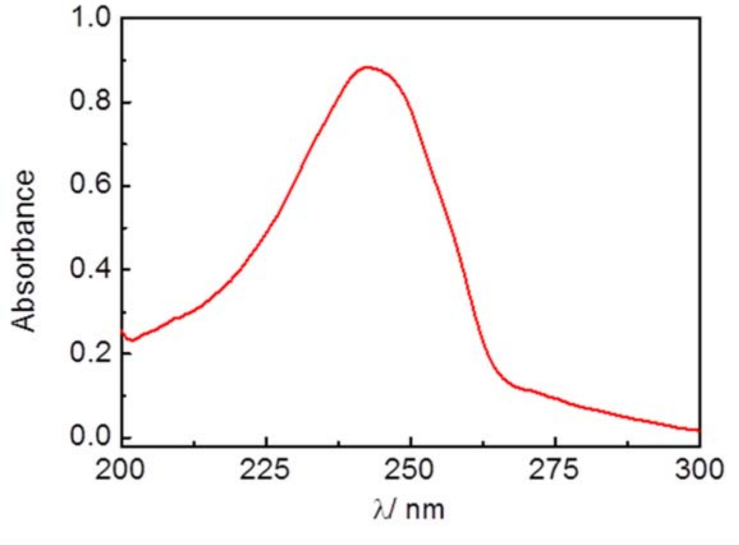

Figure S2. The UV spectrum of HOE_2.

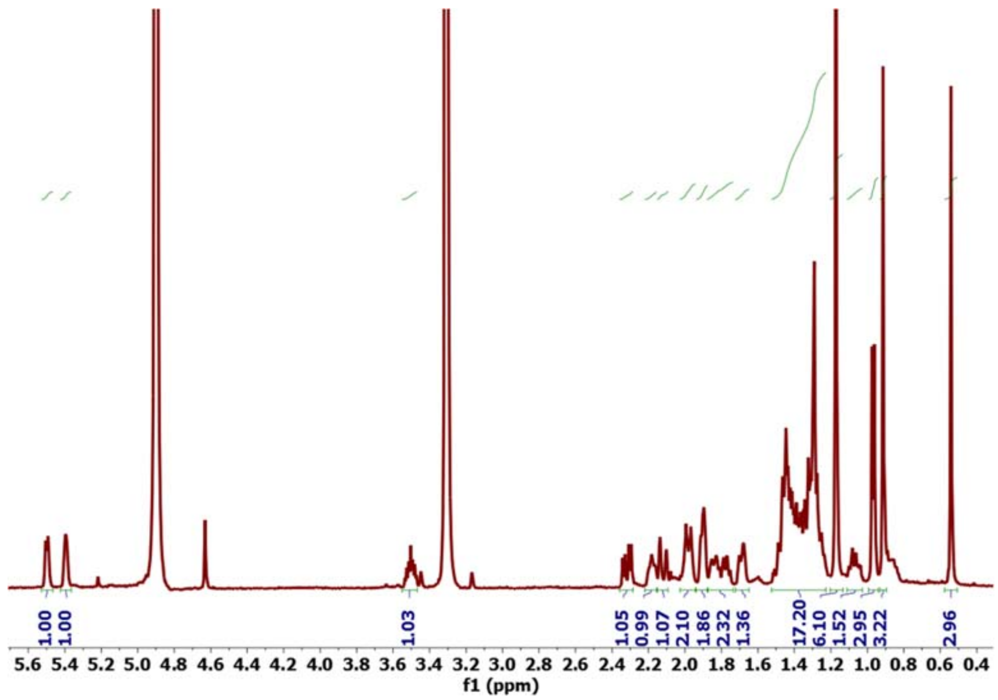

Figure S3. ^{1}H NMR spectrum of HOD_1, in CD_3OD.

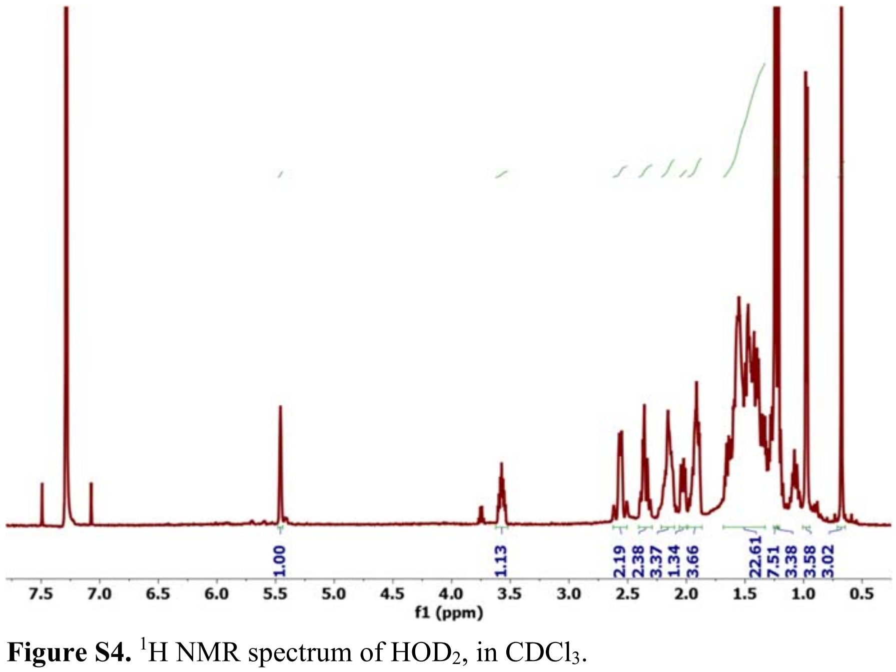

Figure S4. ^{1}H NMR spectrum of HOD_2, in $CDCl_3$.

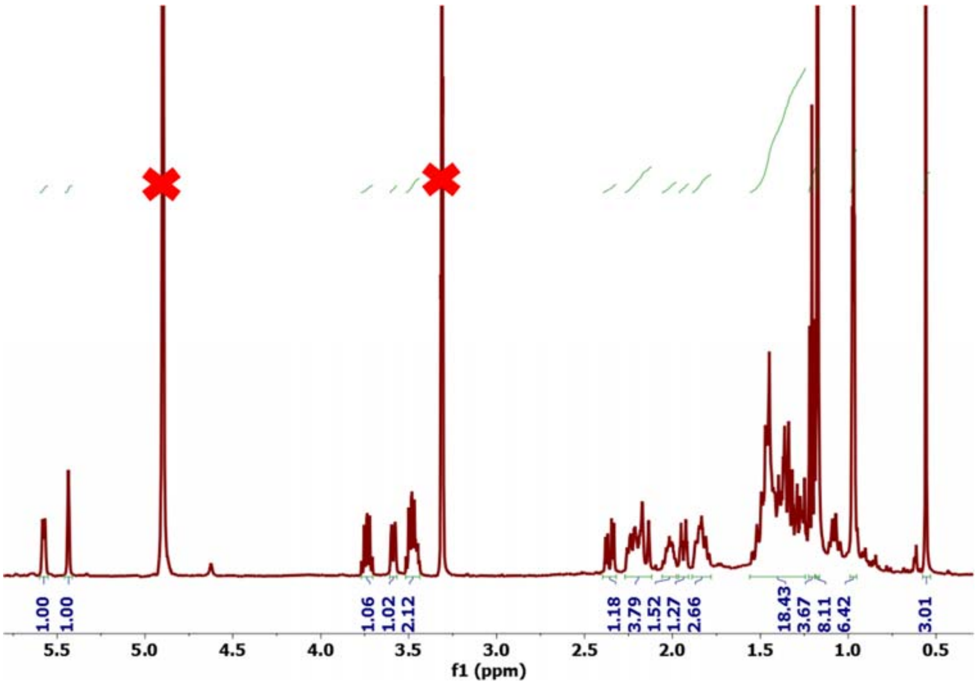

Figure S5. ^{1}H NMR spectrum of HOE_1 in CD_3OD.

.

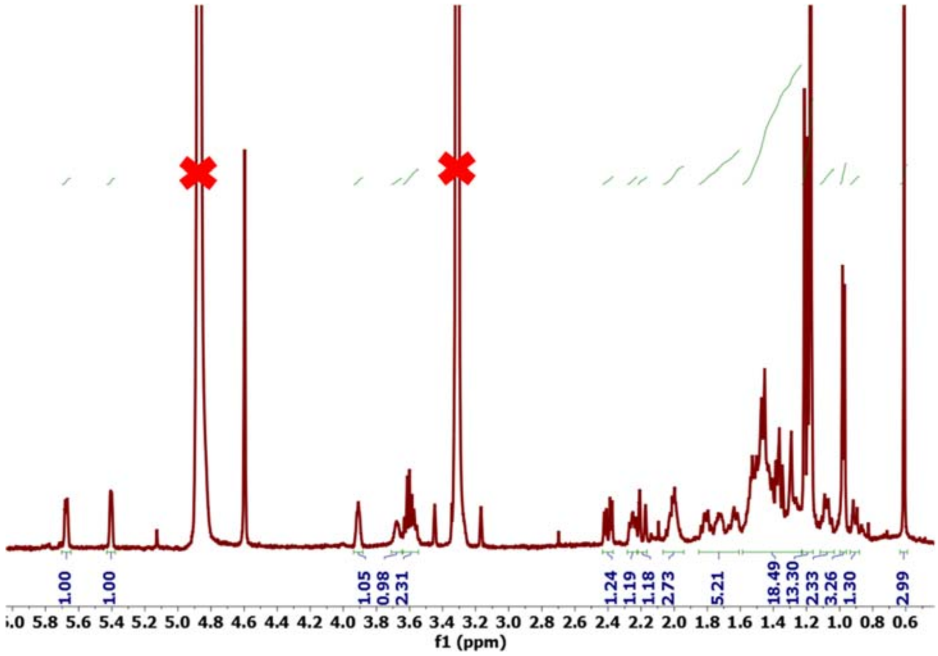

Figure S6. ^{1}H NMR spectrum of HOE_2 in CD_3OD.

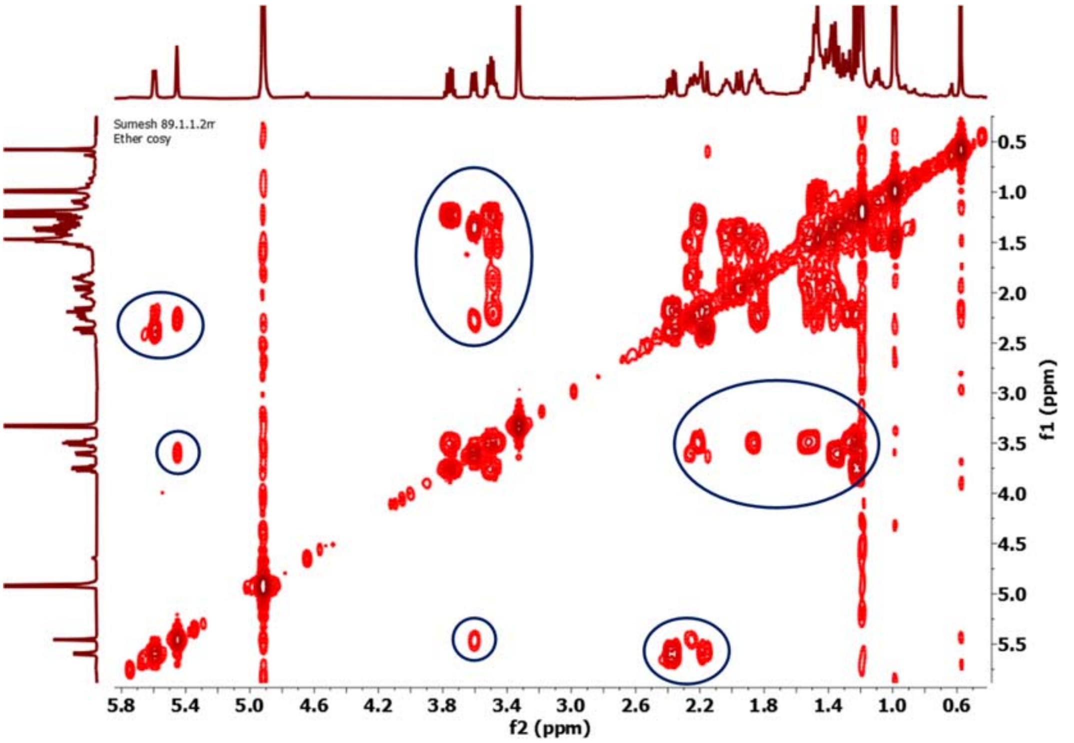

Figure S7. COSY ^{1}H NMR spectrum of HOE_1 in CD_3OD.

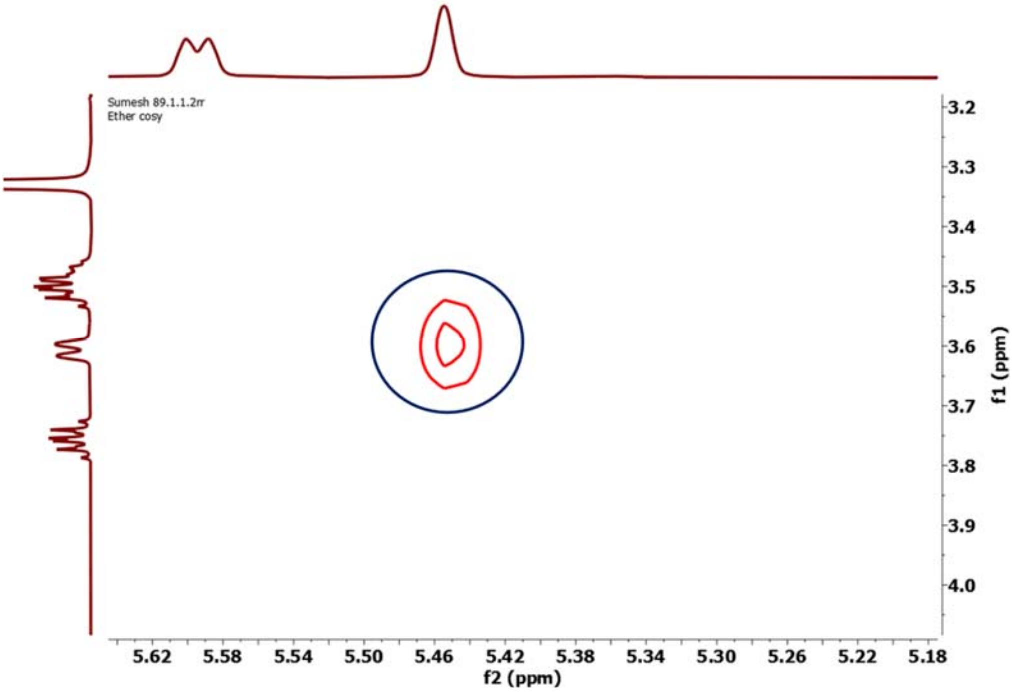

Figure S8. Expanded region of Figure S7 showing the correlation between vinyl H at C_7 and the H at C_6.

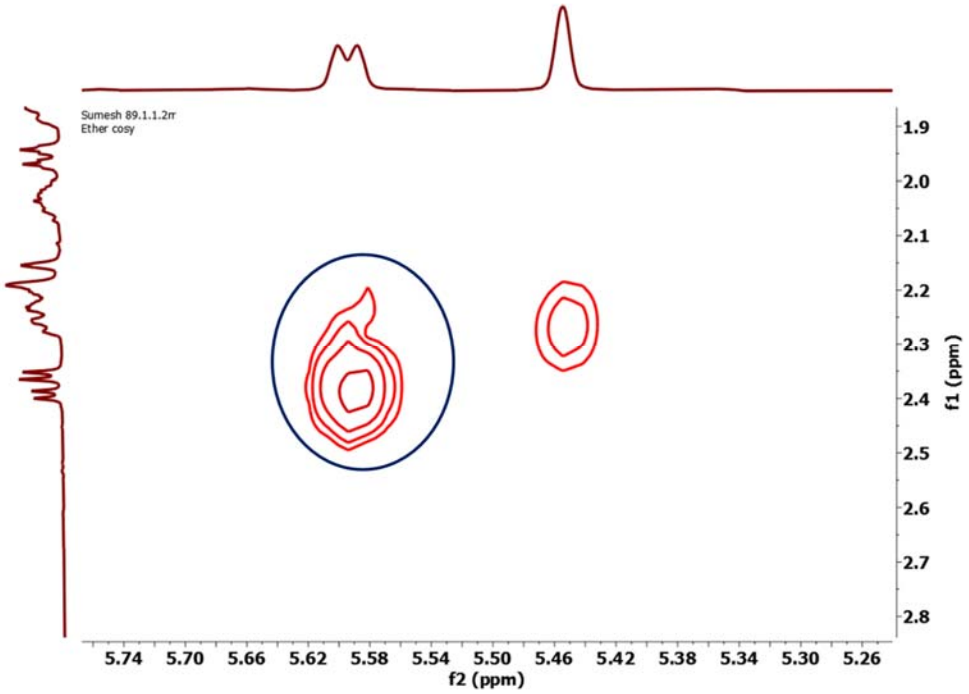

Figure S9. Expanded region of Figure S7 showing the correlation between the vinyl H at C_{11} and the H-6 proton.

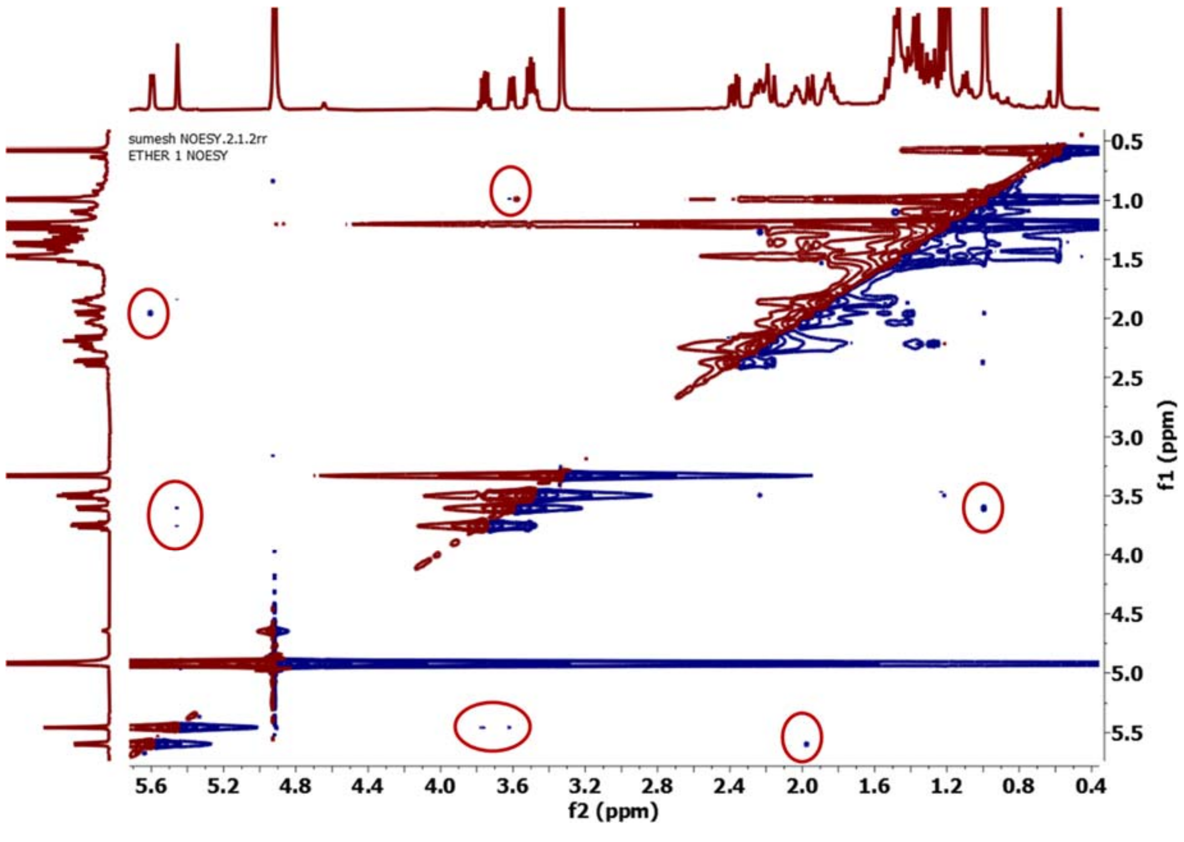

Figure S10. NOESY ^1H NMR spectrum of HOE_1 in CD_3OD.

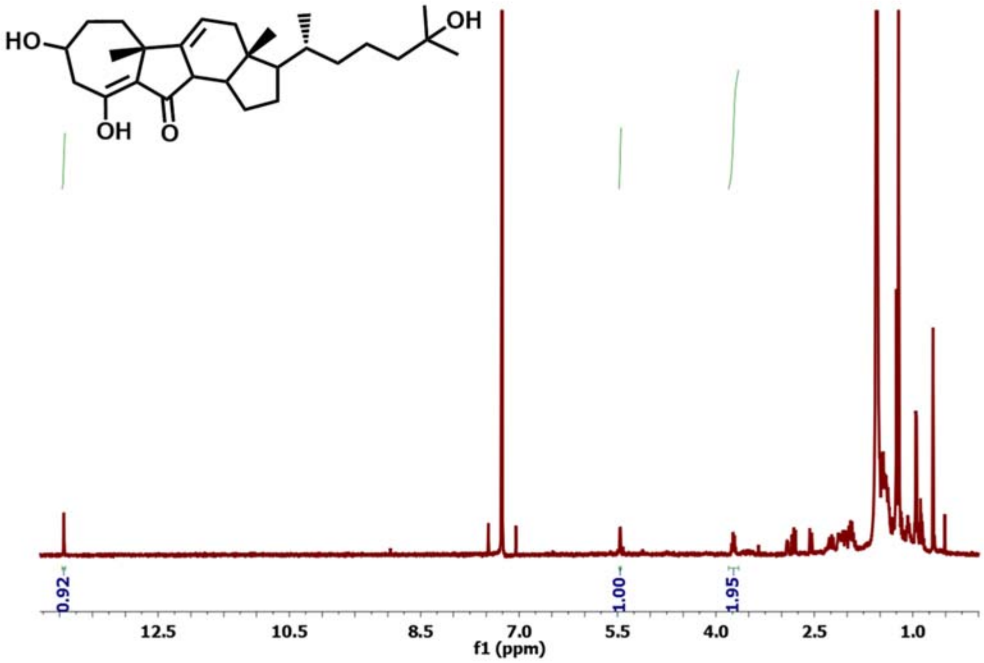

Figure S11. ^1H NMR spectrum of $HORP_2$ in CD_3Cl.

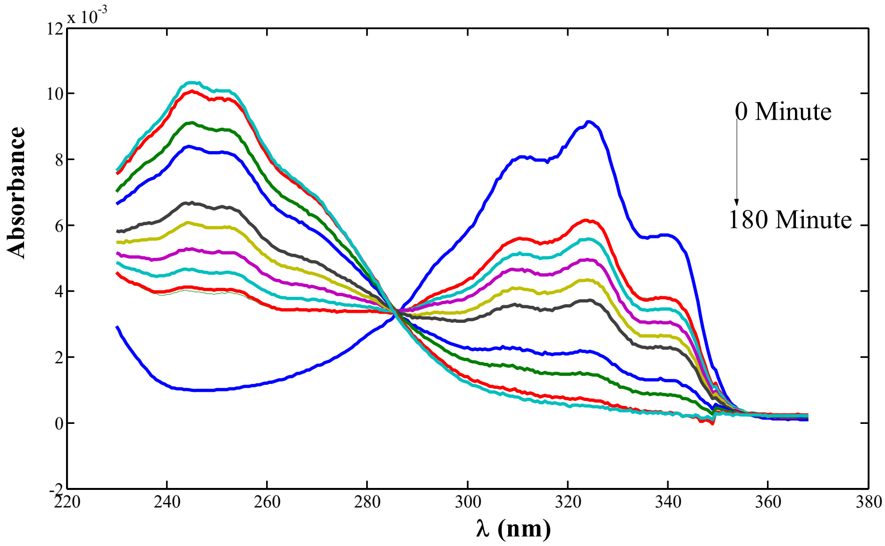

Figure S12. Time evolution of UV spectra from the 313 nm irradiation of CTL in ethanol. PCA of the spectral matrix in Figure S12 revealed a two component system. The combination coefficient plot, Figure S13, shows that the spectral points strictly adhere to the normalization line.

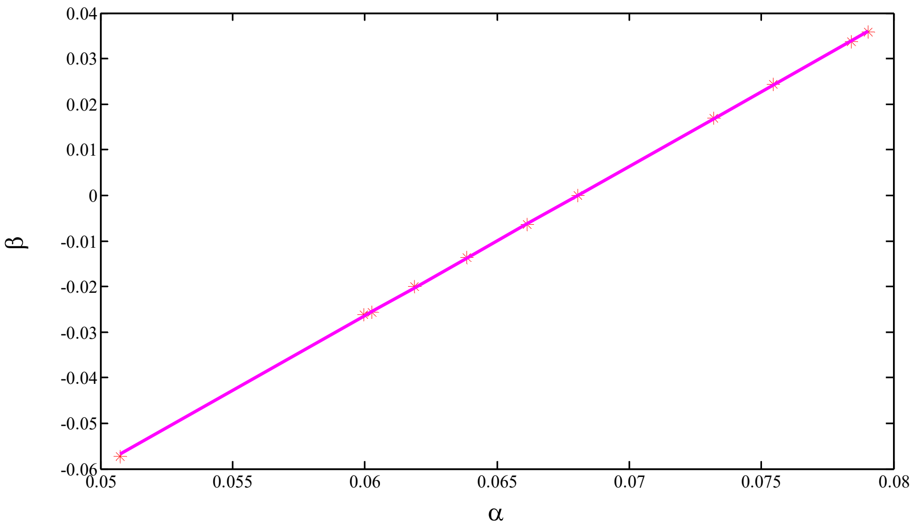

Figure S13. Combination coefficient plot for the spectra in Figure S12.

Crystal Structures

Structural Information

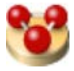

ConDiene123.mol

HOD_1 Structure

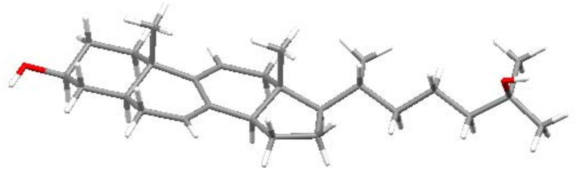

Table S1 Crystal data and structure refinement

Crystal name	HOD_1
Empirical formula	$C_{27}H_{44}O_2$
Formula weight	400.62
Temperature/K	150.00(11)
Crystal system	orthorhombic
Space group	$P2_12_12_1$
a/Å	7.1933(2)
b/Å	9.6267(2)
c/Å	34.9761(9)

α/°	90
β/°	90
γ/°	90
Volume/Å^3	2422.02(10)
Z	4
ρ_{calc}g/cm^3	1.099
μ/mm^{-1}	0.506
F(000)	888.0
Crystal size/mm^3	0.332 × 0.242 × 0.068
Radiation	Cu Kα (λ = 1.54184)
2Θ range for data collection/°	5.054 to 155.784
Index ranges	-9 ≤ h ≤ 9, -9 ≤ k ≤ 12, -44 ≤ l ≤ 43
Reflections collected	18127
Independent reflections	4953 [R_{int} = 0.1169, R_{sigma} = 0.0732]
Data/restraints/parameters	4953/0/270
Goodness-of-fit on F^2	1.061
Final R indexes [I>=2σ (I)]	R_1 = 0.0972, wR_2 = 0.2568
Final R indexes [all data]	R_1 = 0.1073, wR_2 = 0.2643
Largest diff. peak/hole / e Å^{-3}	0.34/-0.32
Flack parameter	0.2(4)

Table S2 Fractional Atomic Coordinates (×10^4) and Equivalent Isotropic Displacement Parameters (Å^2×10^3) for HOD$_1$. U_{eq} is defined as 1/3 of the trace of the orthogonalised U_{IJ} tensor.

**Atom**	***x***	***y***	***Z***	**U(eq)**
C1	2043(8)	8205(6)	824.3(17)	36.9(12)
C2	3594(9)	7205(6)	951.5(17)	37.2(12)
C3	3917(8)	7284(6)	1379.7(16)	35.5(12)
C4	2156(7)	7046(5)	1619.9(15)	29.3(11)
C5	617(8)	8039(6)	1477.9(16)	35.7(12)
C6	290(9)	7940(7)	1044.5(17)	39.5(13)
C7	1495(10)	5527(6)	1595.6(17)	39.7(13)
C8	5518(8)	6400(7)	1506.4(18)	41.8(14)
C9	5825(8)	6454(6)	1923.0(18)	38.8(13)
C10	4497(7)	6912(5)	2172.5(15)	28.6(11)
C11	2647(8)	7305(5)	2045.6(16)	30.2(11)

Atom	*x*	*y*	*z*	U(eq)
C12	1371(9)	7709(6)	2303.6(16)	37.7(13)
C13	1702(8)	7790(6)	2729.0(16)	33.8(12)
C14	3265(7)	6812(5)	2852.2(14)	25.0(10)
C15	4913(8)	7127(6)	2589.3(16)	33.9(12)
C16	2615(9)	5295(5)	2815.2(17)	34.0(12)
C17	6557(8)	6407(7)	2770.4(17)	41.6(14)
C18	6196(9)	6550(7)	3202.9(19)	43.8(15)
C19	4192(8)	7062(5)	3247.9(16)	30.7(11)
C20	3205(8)	6463(6)	3607.8(15)	33.1(12)
C21	1252(9)	6999(8)	3659.3(19)	48.0(15)
C22	4364(10)	6739(6)	3969.9(16)	40.0(14)
C23	3813(12)	5826(6)	4310.2(16)	45.4(16)
C24	4949(16)	6123(6)	4658.5(17)	63(3)
C25	4500(20)	5246(7)	5017.6(19)	84(4)
C26	5660(40)	5714(12)	5342(2)	198(13)
C27	2430(20)	5263(13)	5105(4)	146(9)
O1	1677(7)	8099(5)	425.0(12)	44.6(11)
O2	5018(8)	3842(4)	4924.3(12)	44.4(11)

Table S3 Anisotropic Displacement Parameters ($Å^2 \times 10^3$) for HOD_1. The Anisotropic displacement factor exponent takes the form: $-2\pi^2[h^2a^{*2}U_{11}+2hka^*b^*U_{12}+\ldots]$.

Atom	U_{11}	U_{22}	U_{33}	U_{23}	U_{13}	U_{12}
C1	26(3)	37(3)	48(3)	-3(2)	-2(2)	-5(2)
C2	28(3)	36(3)	47(3)	-1(2)	5(2)	-1(2)
C3	27(3)	34(3)	46(3)	-3(2)	5(2)	5(2)
C4	22(2)	22(2)	43(3)	-1(2)	-1(2)	0(2)
C5	22(2)	39(3)	46(3)	-3(2)	-4(2)	5(2)
C6	31(3)	40(3)	48(3)	-3(2)	-4(2)	5(2)
C7	34(3)	32(3)	53(3)	-5(2)	-2(3)	-11(3)
C8	16(3)	52(4)	58(3)	3(3)	6(2)	8(2)
C9	18(2)	41(3)	58(3)	2(3)	4(2)	9(2)
C10	16(2)	22(2)	48(3)	2.8(19)	4(2)	1.0(18)
C11	22(2)	27(2)	42(3)	-4(2)	0(2)	1(2)

Atom	U_{11}	U_{22}	U_{33}	U_{23}	U_{13}	U_{12}
C12	31(3)	36(3)	46(3)	-1(2)	-4(2)	13(2)
C13	22(3)	29(3)	51(3)	-6(2)	-2(2)	8(2)
C14	19(2)	16(2)	40(3)	1.0(17)	4(2)	-6.6(18)
C15	16(2)	33(3)	52(3)	8(2)	3(2)	-1(2)
C16	34(3)	21(2)	47(3)	0(2)	3(2)	-8(2)
C17	16(2)	56(4)	53(3)	10(3)	-3(2)	2(3)
C18	26(3)	48(4)	57(4)	15(3)	-6(3)	-5(3)
C19	23(2)	20(2)	49(3)	3(2)	-2(2)	-3.9(19)
C20	30(3)	26(2)	42(3)	0(2)	2(2)	-4(2)
C21	32(3)	60(4)	52(3)	-3(3)	4(3)	9(3)
C22	44(3)	32(3)	44(3)	5(2)	0(3)	-5(3)
C23	62(5)	30(3)	44(3)	8(2)	4(3)	-1(3)
C24	123(8)	27(3)	41(3)	8(2)	-2(4)	-12(4)
C25	183(13)	30(3)	38(3)	7(3)	14(5)	18(5)
C26	480(40)	73(7)	38(4)	14(4)	-58(11)	-72(14)
C27	240(20)	91(8)	111(8)	63(7)	114(12)	106(11)
O1	49(3)	42(2)	43(2)	1.2(18)	-3(2)	-2(2)
O2	57(3)	29(2)	47(2)	7.8(16)	4(2)	-0.1(19)

Table S4 Bond Lengths for HOD_1.

Atom	Atom	Length/Å	Atom	Atom	Length/Å
C1	C2	1.539(8)	C13	C14	1.528(7)
C1	C6	1.499(8)	C14	C15	1.531(7)
C1	O1	1.425(7)	C14	C16	1.539(6)
C2	C3	1.517(8)	C14	C19	1.555(7)
C3	C4	1.537(7)	C15	C17	1.510(8)
C3	C8	1.499(8)	C17	C18	1.541(9)
C4	C5	1.545(7)	C18	C19	1.532(8)
C4	C7	1.540(7)	C19	C20	1.556(8)
C4	C11	1.550(8)	C20	C21	1.507(9)
C5	C6	1.537(8)	C20	C22	1.539(8)
C8	C9	1.475(9)	C22	C23	1.532(8)

Atom	Atom	Length/Å	Atom	Atom	Length/Å
C9	C10	1.366(8)	C23	C24	1.494(10)
C10	C11	1.454(7)	C24	C25	1.547(9)
C10	C15	1.503(8)	C25	C26	1.481(18)
C11	C12	1.345(8)	C25	C27	1.52(2)
C12	C13	1.509(8)	C25	O2	1.439(9)

Table S5 Bond Angles for HOD_1.

Atom	Atom	Atom	Angle/°	Atom	Atom	Atom	Angle/°
C6	C1	C2	110.8(5)	C13	C14	C16	109.7(5)
O1	C1	C2	111.9(5)	C13	C14	C19	118.1(4)
O1	C1	C6	109.6(5)	C15	C14	C16	111.9(4)
C3	C2	C1	111.4(5)	C15	C14	C19	99.9(4)
C2	C3	C4	113.9(5)	C16	C14	C19	110.6(4)
C8	C3	C2	112.4(5)	C10	C15	C14	113.7(4)
C8	C3	C4	112.8(5)	C10	C15	C17	120.0(5)
C3	C4	C5	108.8(4)	C17	C15	C14	105.3(4)
C3	C4	C7	111.5(5)	C15	C17	C18	103.8(5)
C3	C4	C11	108.3(5)	C19	C18	C17	106.7(5)
C5	C4	C11	111.8(4)	C14	C19	C20	117.8(4)
C7	C4	C5	110.4(5)	C18	C19	C14	105.2(5)
C7	C4	C11	106.0(4)	C18	C19	C20	113.1(5)
C6	C5	C4	112.9(5)	C21	C20	C19	113.3(5)
C1	C6	C5	111.6(5)	C21	C20	C22	110.3(5)
C9	C8	C3	112.8(5)	C22	C20	C19	110.8(5)
C10	C9	C8	122.5(5)	C23	C22	C20	113.6(5)
C9	C10	C11	121.9(5)	C24	C23	C22	112.5(6)
C9	C10	C15	121.7(5)	C23	C24	C25	116.3(8)
C11	C10	C15	116.3(4)	C26	C25	C24	109.7(10)
C10	C11	C4	117.4(5)	C26	C25	C27	113.4(12)
C12	C11	C4	122.4(5)	C27	C25	C24	111.2(9)
C12	C11	C10	119.7(5)	O2	C25	C24	105.9(6)
C11	C12	C13	124.7(5)	O2	C25	C26	108.2(10)

Atom	Atom	Atom	Angle/°	Atom	Atom	Atom	Angle/°
C12	C13	C14	111.2(4)	O2	C25	C27	108.1(9)
C13	C14	C15	106.2(4)				

Table S6 Torsion Angles for HOD_1.

A	B	C	D	Angle/°	A	B	C	D	Angle/°
C1	C2	C3	C4	54.5(6)	C12	C13	C14	C15	51.2(6)
C1	C2	C3	C8	-175.7(5)	C12	C13	C14	C16	-69.8(6)
C2	C1	C6	C5	55.9(7)	C12	C13	C14	C19	162.2(5)
C2	C3	C4	C5	-52.5(6)	C13	C14	C15	C10	-59.5(5)
C2	C3	C4	C7	69.5(6)	C13	C14	C15	C17	167.2(5)
C2	C3	C4	C11	-174.2(4)	C13	C14	C19	C18	-150.1(5)
C2	C3	C8	C9	-179.2(5)	C13	C14	C19	C20	82.7(6)
C3	C4	C5	C6	52.7(6)	C14	C15	C17	C18	-35.1(6)
C3	C4	C11	C10	-34.7(6)	C14	C19	C20	C21	-58.5(6)
C3	C4	C11	C12	153.6(5)	C14	C19	C20	C22	177.0(5)
C3	C8	C9	C10	18.0(9)	C15	C10	C11	C4	-177.9(5)
C4	C3	C8	C9	-48.7(7)	C15	C10	C11	C12	-6.0(7)
C4	C5	C6	C1	-56.3(7)	C15	C14	C19	C18	-35.7(5)
C4	C11	C12	C13	171.3(5)	C15	C14	C19	C20	-162.9(4)
C5	C4	C11	C10	-154.6(5)	C15	C17	C18	C19	11.6(6)
C5	C4	C11	C12	33.7(7)	C16	C14	C15	C10	60.2(6)
C6	C1	C2	C3	-55.0(6)	C16	C14	C15	C17	-73.1(6)
C7	C4	C5	C6	-70.0(6)	C16	C14	C19	C18	82.3(5)
C7	C4	C11	C10	85.0(6)	C16	C14	C19	C20	-44.9(6)
C7	C4	C11	C12	-86.7(6)	C17	C18	C19	C14	15.5(6)
C8	C3	C4	C5	177.9(5)	C17	C18	C19	C20	145.4(5)
C8	C3	C4	C7	-60.2(7)	C18	C19	C20	C21	178.3(5)
C8	C3	C4	C11	56.1(6)	C18	C19	C20	C22	53.7(6)
C8	C9	C10	C11	3.5(9)	C19	C14	C15	C10	177.3(4)
C8	C9	C10	C15	-172.3(5)	C19	C14	C15	C17	44.0(5)
C9	C10	C11	C4	6.1(7)	C19	C20	C22	C23	-162.5(5)
C9	C10	C11	C12	178.0(6)	C20	C22	C23	C24	-179.5(6)

A	B	C	D	Angle/°	A	B	C	D	Angle/°
C9	C10	C15	C14	-146.5(5)	C21	C20	C22	C23	71.2(7)
C9	C10	C15	C17	-20.7(8)	C22	C23	C24	C25	-179.8(7)
C10	C11	C12	C13	-0.1(9)	C23	C24	C25	C26	-176.9(12)
C10	C15	C17	C18	-164.8(5)	C23	C24	C25	C27	-50.7(11)
C11	C4	C5	C6	172.2(5)	C23	C24	C25	O2	66.5(12)
C11	C10	C15	C14	37.4(6)	O1	C1	C2	C3	-177.6(5)
C11	C10	C15	C17	163.3(5)	O1	C1	C6	C5	179.8(5)
C11	C12	C13	C14	-24.5(8)					

Table S7 Hydrogen Atom Coordinates (Å×10^4) and Isotropic Displacement Parameters (Å^2×10^3) for HOD_1.

Atom	*x*	*Y*	*z*	U(eq)
H1	2459.65	9175.25	879.42	44
H2A	3249.05	6243.32	880.94	45
H2B	4759.58	7442.65	816	45
H3	4302.19	8263.98	1431.79	43
H5A	-556.7	7818.53	1612.66	43
H5B	964.3	9004.74	1543.68	43
H6A	-186.29	7003.25	980.92	47
H6B	-663.43	8627.5	968.11	47
H7A	2365.26	4928.98	1734.74	60
H7B	1444.57	5238.98	1327.04	60
H7C	254.99	5447.27	1709.41	60
H8A	5280.98	5425.62	1429.9	50
H8B	6659.78	6713.86	1374.24	50
H9	6990.16	6159.17	2021.68	47
H12	175.35	7962	2211.5	45
H13A	545.08	7539.73	2865.94	41
H13B	2031.33	8755.18	2799.52	41
H15	5157.62	8143.79	2618.47	41
H16A	3585.27	4674.25	2912.76	51
H16B	2374.18	5081.83	2545.7	51
H16C	1472.99	5162.58	2963.45	51

Atom x	Y	z	U(eq)
H17A 7736.27	6866.14	2697.38	50
H17B 6611.02	5418.27	2693.68	50
H18A 6356.73	5641.58	3331.65	53
H18B 7074.03	7222.67	3317.61	53
H19 4258.99	8091.55	3284.09	37
H20 3124.39	5433.35	3574.24	40
H21A 1265.54	8016.68	3660.09	72
H21B 750.91	6660.66	3902.69	72
H21C 470.76	6667.08	3448.78	72
H22A 5691.99	6578.71	3910.52	48
H22B 4221.16	7726.59	4043.3	48
H23A 2483.85	5980.35	4369.83	54
H23B 3968.4	4837.26	4238.96	54
H24A 4790.1	7115.36	4724.81	76
H24B 6274.37	5980.27	4593.47	76
H26A 5554.56	5049.74	5553.19	297
H26B 5243.65	6631.2	5427.5	297
H26C 6964.55	5770.78	5260.15	297
H27A 1735.11	4928.73	4881.67	220
H27B 2037.1	6214.12	5165.09	220
H27C 2171.61	4658.61	5324.12	220
H1A 2649.73	8279.78	301.86	67
H2 5227.29	3398.03	5126.54	67

HOD_2 Structure

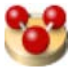

UnconjDiene.mol

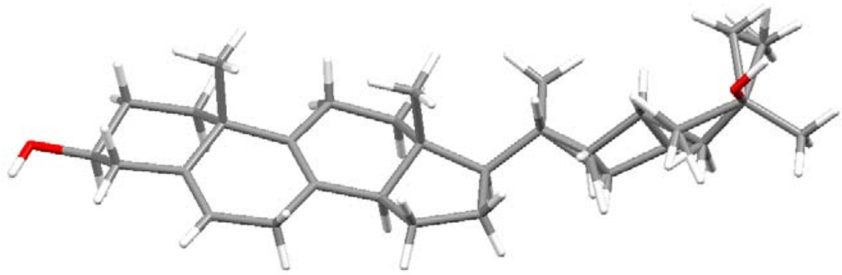

Table S8 Crystal data and structure refinement

Crystal name	HOD_2
Molecular Formula	$C_{27}H_{44}O_2$
Formula Weight	400.62
Temperature/*K*	149.98(10)
Crystal System	Orthorhombic
Space Group	*P*212121
a [Å]	7.33440(10)
b [Å]	9.6312(2)
c [Å]	34.4304(9)
[°]	90
[°]	90
[°]	90
V[$Å^3$]	2432.13(9)
Z	4
ρ_{calc}g/cm^3	1.094
μ/mm^{-1}	0.504
F(000)	888.0
Crystal size/mm^3	0.13 × 0.1 × 0.04
Radiation	Cu Kα (λ = 1.54184)
2*Θ* range for data collection/°	5.134 to 155.072
Index ranges	$-9 \le h \le 9$, $-8 \le k \le 12$, $-43 \le l \le 43$
Reflections collected	24631
Independent reflections	5079 [R_{int} = 0.0329, R_{sigma} = 0.0205]
Data/restraints/parameters	5079/0/321
Goodness-of-fit on F^2	1.198
Final R indexes [I>=2σ (I)]	R_1 = 0.0796, wR_2 = 0.1851
Final R indexes [all data]	R_1 = 0.0847, wR_2 = 0.1877
Largest diff. peak/hole / e $Å^{-3}$	0.23/-0.22
Flack parameter	0.11(2)

Table S9 Fractional Atomic Coordinates (×10^4) and Equivalent Isotropic Displacement Parameters ($Å^2$×10^3) for HOD_2. U_{eq} is defined as 1/3 of of the trace of the orthogonalised U_{IJ} tensor.

**Atom**	***X***	***y***	***z***	**U(eq)**
C1	2710(7)	8334(6)	847.7(17)	53.9(14)
C2	905(7)	8232(6)	1061.1(17)	52.3(14)
C3	1175(6)	8328(5)	1497.3(17)	47.4(13)
C4	2500(6)	7222(5)	1668.9(17)	44.0(12)
C005	4866(6)	7564(5)	2680.0(17)	43.9(12)
C5	4230(6)	7202(5)	1425.8(17)	47.6(13)
C6	3990(7)	7191(6)	992.9(17)	55.8(15)
C7	1602(7)	5763(5)	1641.1(19)	54.2(15)
C8	5879(6)	7132(5)	1583.4(16)	45.1(12)
C9	6211(6)	7070(5)	2005.1(17)	47.4(14)
C10	4558(6)	7379(5)	2252.9(17)	40.2(12)
C11	2891(6)	7510(5)	2098.2(16)	39.2(11)
C12	1254(6)	7843(5)	2349.7(16)	44.8(12)
C13	1655(6)	8137(5)	2778.8(16)	41.4(12)
C14	3196(6)	7217(5)	2933.2(17)	43.0(12)
C15	2681(7)	5675(5)	2890(2)	62.3(18)
C16	6425(6)	6802(6)	2883.2(18)	55.1(15)
C17	5956(7)	6953(6)	3317.4(17)	55.5(15)
C18	3947(6)	7517(5)	3340.2(17)	49.0(13)
C19	2951(7)	6935(7)	3697(2)	73(2)
C20	922(7)	7362(7)	3708(2)	70.0(19)
C21	3936(13)	7691(12)	4122(3)	51(2)
C22	3412(15)	7114(10)	4513(2)	41(2)
C23	4110(20)	6194(14)	4725(4)	54(3)
C24	3526(8)	5055(8)	4989(2)	75(2)
C25	4291(14)	5622(9)	5359(2)	103(3)
C26	1457(19)	5129(16)	5111(4)	58(3)
C27	3777(17)	6928(12)	4001(3)	29(2)
C28	3184(15)	5986(11)	4341(3)	42(2)
C29	4110(16)	5636(13)	4565(3)	39(2)
C30	1423(17)	4674(15)	4919(4)	52(3)

Atom	X	y	z	U(eq)
O1	2401(5)	8234(5)	440.2(12)	62.0(11)
O2	4291(5)	3702(5)	4930.2(12)	60.0(11)

Table S10 Anisotropic Displacement Parameters ($Å^2×10^3$) for HOD_2. The Anisotropic displacement factor exponent takes the form: $-2\pi^2[h^2a^{*2}U_{11}+2hka^*b^*U_{12}+…]$.

Atom	U_{11}	U_{22}	U_{33}	U_{23}	U_{13}	U_{12}
C1	31(2)	59(3)	72(4)	-19(3)	1(2)	-4(2)
C2	29(2)	52(3)	77(4)	-7(3)	-2(3)	3(2)
C3	24(2)	39(3)	78(4)	-8(2)	3(2)	2(2)
C4	21.4(19)	33(2)	77(4)	-7(2)	1(2)	0.6(18)
C005	17.7(18)	33(2)	81(4)	13(2)	-1(2)	-0.5(18)
C5	26(2)	37(3)	80(4)	-19(3)	3(2)	1(2)
C6	29(2)	58(3)	80(4)	-24(3)	0(3)	2(2)
C7	41(3)	39(3)	83(4)	-14(3)	-3(3)	-9(2)
C8	24(2)	42(3)	69(4)	-10(2)	4(2)	1(2)
C9	19(2)	29(2)	94(4)	1(2)	5(2)	3.1(18)
C10	20(2)	27(2)	73(3)	2(2)	4(2)	0.8(17)
C11	22.5(19)	29(2)	66(3)	-6(2)	2(2)	-1.4(17)
C12	21(2)	43(3)	71(4)	-11(2)	1(2)	4(2)
C13	22(2)	32(2)	70(3)	-1(2)	3(2)	3.3(17)
C14	22(2)	27(2)	80(4)	10(2)	7(2)	0.9(17)
C15	33(3)	31(2)	124(6)	8(3)	17(3)	-1(2)
C16	22(2)	57(3)	87(4)	24(3)	0(2)	5(2)
C17	28(2)	61(3)	78(4)	38(3)	5(3)	6(2)
C18	27(2)	43(3)	78(4)	25(3)	5(2)	6(2)
C19	34(3)	81(5)	105(6)	52(4)	24(3)	20(3)
C20	38(3)	86(5)	86(4)	34(4)	17(3)	20(3)
C21	44(5)	69(7)	38(5)	-6(5)	-5(4)	5(5)
C22	64(6)	42(5)	16(4)	-5(4)	3(4)	8(5)
C23	62(8)	56(8)	43(7)	-3(6)	-14(7)	-13(7)
C24	44(3)	78(5)	102(6)	44(4)	5(4)	9(3)
C25	136(8)	78(5)	96(6)	7(5)	17(6)	-16(6)
C26	53(7)	63(9)	58(9)	10(7)	18(7)	19(7)

Atom	U_{11}	U_{22}	U_{33}	U_{23}	U_{13}	U_{12}
C27	44(6)	23(5)	22(5)	0(4)	-10(5)	-3(5)
C28	50(6)	51(6)	23(4)	-2(4)	-5(5)	-6(5)
C29	37(5)	54(7)	26(6)	3(5)	-8(5)	4(5)
C30	36(6)	64(9)	56(9)	-2(6)	11(6)	14(6)
O1	36.3(19)	79(3)	71(3)	-24(2)	1.5(19)	-1(2)
O2	43(2)	77(3)	60(3)	23(2)	8.2(19)	0(2)

Table S11 Bond Lengths for HOD_2.

Atom	Atom	Length/Å	Atom	Atom	Length/Å
C1	C2	1.517(7)	C14	C15	1.540(7)
C1	C6	1.531(8)	C14	C18	1.533(8)
C1	O1	1.425(7)	C16	C17	1.541(8)
C2	C3	1.518(8)	C17	C18	1.572(6)
C3	C4	1.558(7)	C18	C19	1.537(8)
C4	C5	1.520(7)	C19	C20	1.544(7)
C4	C7	1.555(7)	C19	C21	1.785(12)
C4	C11	1.531(8)	C19	C27	1.207(12)
C005	C10	1.498(8)	C21	C22	1.507(12)
C005	C14	1.540(6)	C22	C23	1.256(16)
C005	C16	1.528(6)	C23	C24	1.487(14)
C5	C6	1.501(8)	C24	C25	1.495(11)
C5	C8	1.327(7)	C24	C26	1.577(14)
C8	C9	1.473(8)	C24	C29	1.621(12)
C9	C10	1.512(6)	C24	C30	1.604(14)
C10	C11	1.340(6)	C24	O2	1.433(8)
C11	C12	1.515(6)	C27	C28	1.543(14)
C12	C13	1.533(7)	C28	C29	1.083(14)
C13	C14	1.531(6)			

Table S12 Bond Angles for HOD_2.

Atom	Atom	Atom	Angle/°	Atom	Atom	Atom	Angle/°
C2	C1	C6	109.3(5)	C13	C14	C005	105.4(4)
O1	C1	C2	109.5(4)	C13	C14	C15	110.1(4)
O1	C1	C6	111.7(5)	C13	C14	C18	118.3(4)
C1	C2	C3	111.2(4)	C15	C14	C005	110.5(4)
C2	C3	C4	114.5(4)	C18	C14	C005	101.0(4)
C5	C4	C3	108.7(4)	C18	C14	C15	111.0(5)
C5	C4	C7	107.9(4)	C005	C16	C17	103.4(4)
C5	C4	C11	112.2(4)	C16	C17	C18	106.9(4)
C7	C4	C3	109.3(4)	C14	C18	C17	103.1(4)
C11	C4	C3	111.0(4)	C14	C18	C19	119.5(5)
C11	C4	C7	107.6(5)	C19	C18	C17	111.1(4)
C10	C005	C14	114.2(4)	C18	C19	C20	112.3(5)
C10	C005	C16	120.3(4)	C27	C19	C18	117.1(8)
C16	C005	C14	103.4(4)	C27	C19	C20	117.7(9)
C6	C5	C4	116.7(4)	C22	C21	C19	118.6(8)
C8	C5	C4	122.4(5)	C23	C22	C21	132.5(11)
C8	C5	C6	120.8(5)	C22	C23	C24	139.4(13)
C5	C6	C1	113.0(4)	C23	C24	C26	113.9(9)
C5	C8	C9	123.7(5)	C25	C24	C29	122.8(7)
C8	C9	C10	114.6(4)	C25	C24	C30	124.9(8)
C005	C10	C9	117.2(4)	C30	C24	C29	101.4(8)
C11	C10	C005	121.1(4)	O2	C24	C25	107.8(6)
C11	C10	C9	121.7(5)	O2	C24	C29	94.8(7)
C10	C11	C4	122.5(4)	O2	C24	C30	98.5(7)
C10	C11	C12	121.1(5)	C19	C27	C28	121.2(10)
C12	C11	C4	116.2(4)	C29	C28	C27	123.2(11)
C11	C12	C13	116.0(4)	C28	C29	C24	125.8(11)
C14	C13	C12	111.7(4)				

Table S13 Torsion Angles for HOD_2.

A	B	C	D	Angle/°	A	B	C	D	Angle/°
C1	C2	C3	C4	-56.6(6)	C11	C4	C5	C6	-169.1(4)
C2	C1	C6	C5	-54.2(6)	C11	C4	C5	C8	14.0(7)
C2	C3	C4	C5	48.6(5)	C11	C12	C13	C14	-36.6(6)
C2	C3	C4	C7	-69.0(6)	C12	C13	C14	C005	59.8(5)
C2	C3	C4	C11	172.5(4)	C12	C13	C14	C15	-59.4(6)
C3	C4	C5	C6	-45.9(6)	C12	C13	C14	C18	171.6(4)
C3	C4	C5	C8	137.2(5)	C13	C14	C18	C17	-153.4(4)
C3	C4	C11	C10	-138.7(5)	C13	C14	C18	C19	82.9(6)
C3	C4	C11	C12	46.0(5)	C14	C005	C10	C9	-153.7(4)
C4	C5	C6	C1	51.0(6)	C14	C005	C10	C11	27.6(6)
C4	C5	C8	C9	-0.2(8)	C14	C005	C16	C17	-36.6(6)
C4	C11	C12	C13	-179.7(4)	C14	C18	C19	C20	-55.0(8)
C005	C10	C11	C4	-175.3(4)	C14	C18	C19	C27	164.2(8)
C005	C10	C11	C12	-0.3(7)	C15	C14	C18	C17	78.0(5)
C005	C14	C18	C17	-39.2(4)	C15	C14	C18	C19	-45.7(6)
C005	C14	C18	C19	-162.8(4)	C16	C005	C10	C9	-29.8(6)
C005	C16	C17	C18	11.7(6)	C16	C005	C10	C11	151.5(5)
C5	C4	C11	C10	-16.9(6)	C16	C005	C14	C13	171.5(4)
C5	C4	C11	C12	167.9(4)	C16	C005	C14	C15	-69.7(6)
C5	C8	C9	C10	-11.6(7)	C16	C005	C14	C18	47.8(5)
C6	C1	C2	C3	57.0(6)	C16	C17	C18	C14	17.3(5)
C6	C5	C8	C9	-177.0(5)	C16	C17	C18	C19	146.4(6)
C7	C4	C5	C6	72.5(6)	C17	C18	C19	C20	-174.7(6)
C7	C4	C5	C8	-104.4(6)	C17	C18	C19	C27	44.5(10)
C7	C4	C11	C10	101.7(5)	C18	C19	C27	C28	-160.9(8)
C7	C4	C11	C12	-73.5(5)	C19	C21	C22	C23	92.5(15)
C8	C5	C6	C1	-132.0(5)	C19	C27	C28	C29	155.3(13)
C8	C9	C10	C005	-170.2(4)	C20	C19	C27	C28	60.4(13)
C8	C9	C10	C11	8.5(7)	C21	C22	C23	C24	-142.1(16)
C9	C10	C11	C4	6.0(7)	C22	C23	C24	C26	-15(2)
C9	C10	C11	C12	-179.0(4)	C25	C24	C29	C28	-127.2(13)

A	B	C	D	Angle/°	A	B	C	D	Angle/°
C10	C005	C14	C13	-56.0(5)	C27	C28	C29	C24	160.6(10)
C10	C005	C14	C15	62.9(6)	C30	C24	C29	C28	18.3(16)
C10	C005	C14	C18	-179.6(4)	O1	C1	C2	C3	179.7(4)
C10	C005	C16	C17	-165.4(4)	O1	C1	C6	C5	-175.6(4)
C10	C11	C12	C13	5.0(7)	O2	C24	C29	C28	118.0(14)

Table S14 Hydrogen Atom Coordinates (Å×10^4) and Isotropic Displacement Parameters (Å^2×10^3) for HOD_2.

**Atom**	***x***	***Y***	***z***	**U(eq)**
H1	3260.87	9238.57	904.25	65
H2A	322.7	7356.66	997.97	63
H2B	108.21	8976.25	976.12	63
H3A	-2.08	8230.02	1622.92	57
H3B	1638.33	9244.7	1559.5	57
H005	5100.98	8556.45	2718.36	53
H6A	3508.95	6295.74	914.46	67
H6B	5172.19	7305.3	870.67	67
H7A	2421.5	5077.67	1743.83	81
H7B	1340.78	5553.41	1374.23	81
H7C	489.83	5756.32	1788.04	81
H8	6882.17	7120.63	1418.16	54
H9A	7164.06	7728.55	2069.89	57
H9B	6655.48	6150.17	2069.97	57
H12A	410.26	7068.79	2335.26	54
H12B	643.41	8646.66	2240.79	54
H13A	1991.37	9104.47	2809.2	50
H13B	560.78	7973.5	2930.44	50
H15A	2537.17	5455.37	2619.88	93
H15B	1556.86	5500.84	3024.29	93
H15C	3627.98	5108.66	2999.24	93
H16A	7590.25	7229.13	2824.24	66
H16B	6462.69	5832.86	2806.96	66
H17A	6789.93	7596.11	3441.77	67

**Atom**	***x***	***Y***	***z***	**U(eq)**
H17B	6047.52	6061.46	3447.13	67
H18	4014.4	8527.98	3370.31	59
H19	2925	5792.4	3660(20)	100(30)
H20A	828.79	8355.29	3698.72	105
H20B	374.01	7025.73	3942.86	105
H20C	302.59	6969	3488.08	105
H21A	5250.4	7619.18	4097.64	61
H21B	3637.02	8671.75	4120.89	61
H22A	2372.05	7519.19	4618.29	49
H22	3880(90)	7480(70)	4787(18)	80(20)
H23	5372.69	6237.74	4712.83	64
H25A	3969.61	5019.99	5570.46	155
H25B	3800.62	6531.06	5405.08	155
H25C	5594.65	5678.34	5339.01	155
H26A	709.01	4811.65	4900.53	87
H26B	1143.38	6070.77	5173.84	87
H26C	1258.39	4550.19	5334.43	87
H27A	4789.75	7499.4	4029.89	35
H27	4616.3	6165.9	3950	160(100)
H28A	1971.87	5706.06	4353.33	50
H28	3540(90)	4910(80)	4290(19)	80(20)
H29	5349.72	5671.06	4509.74	47
H30A	1309.69	4130.58	4686.47	78
H30B	727.07	5512.77	4892.43	78
H30C	973.4	4150.59	5136.08	78
H1A	3375.65	8295.82	324.89	93
H2	3860(100)	3080(80)	5120(20)	90(20)

Table S15 Atomic Occupancy for HOD_2.

**Atom**	***Occupancy***	**Atom**	***Occupancy***	**Atom**	***Occupancy***
C21	0.6	H21A	0.6	H21B	0.6
C22	0.5	H22A	0.5	C23	0.5
H23	0.5	C26	0.5	H26A	0.5
H26B	0.5	H26C	0.5	C27	0.4
H27A	0.4	H27	0.4	C28	0.5
H28A	0.5	C29	0.5	H29	0.5
C30	0.5	H30A	0.5	H30B	0.5
H30C	0.5				

Rearranged peroxide product $HORP_2$

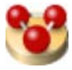

s-7r-1123.cif

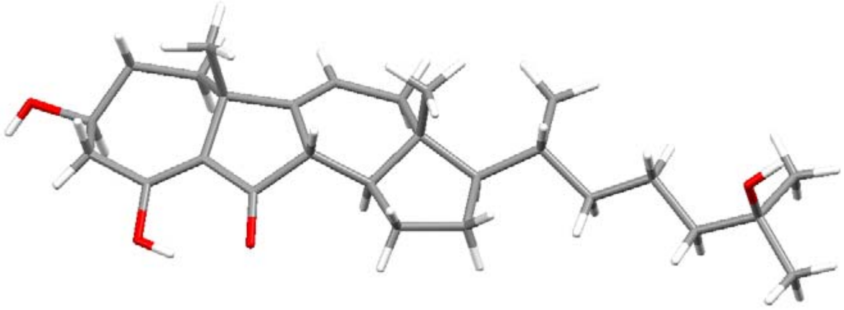

Crystallography. Single clear plate-shaped crystal of approximate dimension 0.60 × 0.07 × 0.03 mm^3 was adhered to a Mitegen loop with Paratone oil. Crystallographic data were collected at temperature 149.99 K on a Rigaku-Oxford Diffraction XtaLAB-Synergy-S diffractometer with a Hypix-6000HE (Hybrid Photon Counting) detector, using Cu-Kα radiation of wavelength 1.54187 Å. The intensity data were measured by a ω-scan with 0.5° oscillations for each frame with the intensity that is more than 10:1 for data-to-parameter ratio. The program suite CrysAlis^{Pro} was used for data collection, absorption correction, and data reduction. The structures were solved with the dual-space algorithm using SHELXT and were refined by full-matrix least-squares methods on F^2 with SHELXL-2014 using the GUI Olex2 [1]. The compound $C_{27}H_{42}O_4$ crystallized in the orthorhombic crystal system, with the space group $P2_12_12_1$. All non-hydrogen atoms were refined anisotropically. Hydrogen atoms were inserted at calculated positions or if possible based on difference Fourier analysis and refined with a riding model or without restrictions.

Table S16 Crystal data and structure refinement

Crystal name	$HORP_2$
Molecular Formula	$C_{27}H_{42}O_4$
Formula Weight	430.60
Temperature [*K*]	149.99(10)
Crystal System	orthorhombic
Space Group	$P2_12_12_1$
a [Å]	7.52147(13)
b [Å]	9.40295(17)
c [Å]	35.0317(6)
[°]	90
[°]	90
[°]	90
V[Å^3]	2477.59(8)
Z	4
ρ_{calc} [g/cm^3]	1.154
μ/mm^{-1}	0.593
F(000)	944.0
Crystal size [mm^3]	0.596 × 0.069 × 0.029
Radiation	Cu Kα (λ = 1.54184)
2Θ range for data collection [°]	5.046 to 160.658
Index ranges	$-9 \leq h \leq 9$, $-9 \leq k \leq 11$, $-44 \leq l \leq 38$
Reflections collected	25536
Independent reflections	5225 [R_{int} = 0.0647, R_{sigma} = 0.0443]
Data/restraints/parameters	5225/0/289
Goodness-of-fit on F^2	1.060
Final R indexes [I>=2σ (I)]	$R1$ = 0.0511, wR_2 = 0.1308
Final R indexes [all data]	$R1$ = 0.0563, wR_2 = 0.1375
Largest diff. peak/hole / e Å^{-3}	0.36/-0.22
Flack parameter	0.2(3)

Table S17 Fractional Atomic Coordinates (×10[4]) and Equivalent Isotropic Displacement Parameters (Å[2]×10[3]) for $HORP_2$. U_{eq} is defined as 1/3 of of the trace of the orthogonalised U_{IJ} tensor.

**Atom**	***X***	***y***	***z***	**U(eq)**
O1	8570(3)	2325(2)	473.7(5)	36.3(5)
O2	3574(2)	3765(3)	1142.2(6)	42.7(5)
O4	4127(3)	6205(2)	4966.5(5)	34.9(4)
C1	7874(4)	2477(3)	851.6(8)	31.5(6)
C2	9458(3)	2857(3)	1101.9(8)	29.1(5)
C3	9110(3)	2889(3)	1533.3(7)	26.8(5)
C4	7934(3)	4091(3)	1685.4(7)	25.6(5)
C5	5995(3)	4022(3)	1555.7(7)	26.7(5)
C6	5338(3)	3783(3)	1201.0(8)	31.0(6)
C7	6376(4)	3573(4)	840.5(8)	35.9(6)
C8	8749(4)	5557(3)	1588.8(8)	32.1(6)
C9	7676(3)	4000(3)	2118.5(7)	25.6(5)
C10	5886(3)	4630(3)	2221.6(7)	25.5(5)
C11	4808(3)	4326(3)	1867.1(8)	26.7(5)
C12	8694(3)	3350(3)	2376.0(8)	30.1(5)
C13	8222(3)	3213(3)	2794.6(8)	31.6(6)
C14	6658(3)	4167(3)	2911.7(7)	25.5(5)
C15	5212(3)	4044(3)	2598.4(7)	25.5(5)
C16	3545(4)	4708(3)	2770.9(8)	34.0(6)
C17	3713(4)	4392(3)	3201.7(8)	32.0(6)
C18	5567(3)	3701(3)	3268.6(8)	27.4(5)
C19	7303(4)	5703(3)	2955.2(9)	36.4(6)
C20	8130(5)	3402(5)	3744.4(9)	53.6(10)
C21	6298(4)	4059(3)	3667.7(7)	33.3(6)
C22	4972(4)	3594(3)	3977.6(8)	36.3(6)
C23	5292(4)	4280(3)	4368.0(8)	34.7(6)
C24	3736(4)	4019(3)	4635.6(8)	33.8(6)
C25	3875(4)	4710(3)	5031.6(8)	33.1(6)
C26	5415(5)	4099(3)	5258.6(9)	41.9(7)
C27	2124(4)	4498(4)	5247.8(9)	43.2(7)
O3	3156(2)	4374(2)	1849.0(6)	35.5(5)

Table S18 Anisotropic Displacement Parameters ($Å^2×10^3$) for $HORP_2$. The Anisotropic displacement factor exponent takes the form: $-2\pi^2[h^2a^{*2}U_{11}+2hka^*b^*U_{12}+\dots]$.

**Atom**	**U_{11}**	**U_{22}**	**U_{33}**	**U_{23}**	**U_{13}**	**U_{12}**
O1	28.7(9)	53.9(12)	26.3(9)	-4.5(9)	0.3(8)	-7.7(9)
O2	15.7(8)	82.4(16)	30.1(10)	2.4(11)	-5.3(8)	-1.0(9)
O4	38.7(10)	37.5(10)	28.7(9)	-4.0(8)	-7.7(8)	2.5(8)
C1	25.9(12)	42.3(14)	26.2(13)	-0.8(11)	-1.3(10)	-5.7(11)
C2	19.8(11)	36.9(13)	30.5(13)	-0.9(11)	0.0(10)	-1.2(10)
C3	19.6(11)	31.1(12)	29.7(13)	0.7(10)	-1.0(9)	0.1(9)
C4	16.4(10)	33.1(12)	27.5(12)	1.8(10)	-2.9(9)	0.5(9)
C5	17.6(10)	32.2(12)	30.3(13)	4.3(10)	-0.7(9)	-1.2(9)
C6	16.4(11)	44.8(15)	31.8(14)	5.6(12)	-2.5(10)	-1.5(10)
C7	24.2(12)	58.6(18)	24.9(12)	2.7(12)	-2.5(11)	-0.7(12)
C8	25.7(12)	35.8(13)	34.9(14)	2.1(11)	1.0(11)	-3.5(11)
C9	18.3(11)	29.2(11)	29.4(13)	-2.0(10)	0.5(9)	-0.9(9)
C10	19.3(11)	27.5(11)	29.7(12)	2.0(10)	0.1(10)	0.8(9)
C11	19.5(11)	29.7(12)	30.9(13)	4.2(10)	-1.2(10)	0.7(9)
C12	19.5(11)	42.0(14)	28.7(12)	-2.1(11)	-1.7(10)	4.0(10)
C13	22.0(11)	45.9(15)	27.0(13)	0.4(11)	-1.8(10)	8.8(11)
C14	20.1(11)	29.9(12)	26.5(12)	-2.3(10)	-1.6(9)	2.0(9)
C15	17.7(10)	29.9(12)	29.0(13)	-0.1(10)	-1.3(9)	-0.8(9)
C16	19.9(11)	47.3(15)	34.8(14)	-0.5(12)	2.2(11)	5.4(11)
C17	23.2(12)	38.0(13)	34.8(14)	-2.5(11)	3.1(10)	2.0(11)
C18	25.5(12)	28.0(11)	28.6(12)	-3.2(10)	1.6(10)	1.4(10)
C19	34.8(14)	38.8(14)	35.6(15)	-3.2(12)	-1.0(12)	-8.2(12)
C20	36.8(16)	95(3)	28.7(14)	-4.3(16)	-2.5(13)	20.3(18)
C21	30.4(13)	44.5(15)	24.9(12)	-3.8(11)	1.1(11)	0.6(12)
C22	38.2(15)	41.2(15)	29.6(14)	-3.4(12)	0.2(12)	-1.1(12)
C23	34.5(14)	41.1(15)	28.5(13)	-2.4(11)	1.0(11)	-0.1(12)
C24	33.0(13)	40.9(14)	27.6(13)	-2.9(11)	-0.9(11)	-1.0(12)
C25	34.4(14)	39.1(14)	25.9(13)	-1.8(11)	0.8(11)	1.8(11)
C26	50.8(17)	42.3(15)	32.5(15)	-2.6(13)	-8.4(13)	9.7(14)
C27	44.0(17)	51.7(17)	33.8(15)	-5.8(14)	9.1(13)	-2.2(14)
O3	16.3(8)	55.0(12)	35.3(10)	4.0(9)	-1.2(7)	4.0(8)

Table S19 Bond Lengths for $HORP_2$.

Atom	Atom	Length/Å	Atom	Atom	Length/Å
O1	C1	1.431(3)	C11	O3	1.245(3)
O2	C6	1.342(3)	C12	C13	1.515(4)
O4	C25	1.437(4)	C13	C14	1.535(3)
C1	C2	1.522(4)	C14	C15	1.550(3)
C1	C7	1.528(4)	C14	C18	1.559(4)
C2	C3	1.534(4)	C14	C19	1.531(4)
C3	C4	1.531(4)	C15	C16	1.526(4)
C4	C5	1.529(3)	C16	C17	1.543(4)
C4	C8	1.546(4)	C17	C18	1.556(4)
C4	C9	1.532(4)	C18	C21	1.540(4)
C5	C6	1.356(4)	C20	C21	1.534(4)
C5	C11	1.439(4)	C21	C22	1.538(4)
C6	C7	1.498(4)	C22	C23	1.531(4)
C9	C10	1.514(3)	C23	C24	1.519(4)
C9	C12	1.331(4)	C24	C25	1.535(4)
C10	C11	1.510(4)	C25	C26	1.517(4)
C10	C15	1.517(4)	C25	C27	1.532(4)

Table S20 Bond Angles for $HORP_2$.

Atom	Atom	Atom	Angle/˚	Atom	Atom	Atom	Angle/˚
O1	C1	C2	105.7(2)	C12	C13	C14	112.8(2)
O1	C1	C7	108.3(2)	C13	C14	C15	107.8(2)
C2	C1	C7	115.7(2)	C13	C14	C18	117.0(2)
C1	C2	C3	116.0(2)	C15	C14	C18	100.22(19)
C4	C3	C2	117.1(2)	C19	C14	C13	109.6(2)
C3	C4	C8	110.7(2)	C19	C14	C15	111.3(2)
C3	C4	C9	112.1(2)	C19	C14	C18	110.6(2)
C5	C4	C3	114.6(2)	C10	C15	C14	110.8(2)
C5	C4	C8	110.6(2)	C10	C15	C16	118.1(2)
C5	C4	C9	99.85(19)	C16	C15	C14	105.4(2)
C9	C4	C8	108.5(2)	C15	C16	C17	104.0(2)
C6	C5	C4	128.9(2)	C16	C17	C18	107.5(2)
C6	C5	C11	120.1(2)	C17	C18	C14	103.5(2)
C11	C5	C4	111.0(2)	C21	C18	C14	118.6(2)
O2	C6	C5	120.2(2)	C21	C18	C17	111.5(2)
O2	C6	C7	112.6(2)	C20	C21	C18	113.1(2)
C5	C6	C7	127.2(2)	C20	C21	C22	110.2(3)
C6	C7	C1	116.9(2)	C22	C21	C18	110.3(2)
C10	C9	C4	109.1(2)	C23	C22	C21	114.2(2)
C12	C9	C4	128.6(2)	C24	C23	C22	111.3(2)
C12	C9	C10	122.0(2)	C23	C24	C25	115.9(2)
C9	C10	C15	111.2(2)	O4	C25	C24	106.2(2)
C11	C10	C9	102.0(2)	O4	C25	C26	110.7(2)
C11	C10	C15	117.9(2)	O4	C25	C27	108.6(2)
C5	C11	C10	109.1(2)	C26	C25	C24	111.4(2)
O3	C11	C5	126.0(3)	C26	C25	C27	110.3(3)
O3	C11	C10	124.8(2)	C27	C25	C24	109.4(2)
C9	C12	C13	124.1(2)				

Table S21 Torsion Angles for $HORP_2$.

A	B	C	D	Angle/°	A	B	C	D	Angle/°
O1	C1	C2	C3	-172.2(2)	C11	C5	C6	O2	-3.1(4)
O1	C1	C7	C6	173.9(2)	C11	C5	C6	C7	174.5(3)
O2	C6	C7	C1	-132.0(3)	C11	C10	C15	C14	167.7(2)
C1	C2	C3	C4	-69.7(3)	C11	C10	C15	C16	-70.7(3)
C2	C1	C7	C6	-67.7(3)	C12	C9	C10	C11	-143.7(3)
C2	C3	C4	C5	66.7(3)	C12	C9	C10	C15	-17.2(3)
C2	C3	C4	C8	-59.2(3)	C12	C13	C14	C15	43.8(3)
C2	C3	C4	C9	179.6(2)	C12	C13	C14	C18	155.6(2)
C3	C4	C5	C6	-46.1(4)	C12	C13	C14	C19	-77.5(3)
C3	C4	C5	C11	137.0(2)	C13	C14	C15	C10	-64.5(3)
C3	C4	C9	C10	-150.5(2)	C13	C14	C15	C16	166.7(2)
C3	C4	C9	C12	22.3(4)	C13	C14	C18	C17	-154.8(2)
C4	C5	C6	O2	-179.8(3)	C13	C14	C18	C21	81.1(3)
C4	C5	C6	C7	-2.2(5)	C14	C15	C16	C17	-31.2(3)
C4	C5	C11	C10	0.5(3)	C14	C18	C21	C20	-59.7(3)
C4	C5	C11	O3	178.7(3)	C14	C18	C21	C22	176.4(2)
C4	C9	C10	C11	29.7(2)	C15	C10	C11	C5	-140.2(2)
C4	C9	C10	C15	156.2(2)	C15	C10	C11	O3	41.6(4)
C4	C9	C12	C13	-174.1(2)	C15	C14	C18	C17	-38.8(2)
C5	C4	C9	C10	-28.8(2)	C15	C14	C18	C21	-162.8(2)
C5	C4	C9	C12	144.1(3)	C15	C16	C17	C18	6.0(3)
C5	C6	C7	C1	50.3(4)	C16	C17	C18	C14	21.0(3)
C6	C5	C11	C10	-176.7(2)	C16	C17	C18	C21	149.5(2)
C6	C5	C11	O3	1.4(4)	C17	C18	C21	C20	-179.7(3)
C7	C1	C2	C3	67.9(3)	C17	C18	C21	C22	56.4(3)
C8	C4	C5	C6	79.8(3)	C18	C14	C15	C10	172.8(2)
C8	C4	C5	C11	-97.2(3)	C18	C14	C15	C16	43.9(2)
C8	C4	C9	C10	87.0(2)	C18	C21	C22	C23	-162.7(3)
C8	C4	C9	C12	-100.2(3)	C19	C14	C15	C10	55.7(3)
C9	C4	C5	C6	-166.1(3)	C19	C14	C15	C16	-73.1(3)
C9	C4	C5	C11	17.0(3)	C19	C14	C18	C17	78.8(3)

A	B	C	D	Angle/°	A	B	C	D	Angle/°
C9	C10	C11	C5	-18.1(3)	C19	C14	C18	C21	-45.3(3)
C9	C10	C11	O3	163.7(3)	C20	C21	C22	C23	71.7(3)
C9	C10	C15	C14	50.5(3)	C21	C22	C23	C24	168.3(3)
C9	C10	C15	C16	172.1(2)	C22	C23	C24	C25	-177.7(2)
C9	C12	C13	C14	-12.4(4)	C23	C24	C25	O4	56.3(3)
C10	C9	C12	C13	-2.1(4)	C23	C24	C25	C26	-64.3(3)
C10	C15	C16	C17	-155.6(2)	C23	C24	C25	C27	173.4(3)

Table S22 Hydrogen Atom Coordinates (Å×10^4) and Isotropic Displacement Parameters (Å^2×10^3) for HORP$_2$.

Atom	*x*	*y*	*z*	U(eq)
H1	7828	1894	337	54
H2	3047	3980	1346	64
H4	4589	6577	5161	52
H1A	7379	1543	937	38
H2A	9899	3805	1023	35
H2B	10419	2163	1051	35
H3A	8556	1973	1606	32
H3B	10273	2944	1665	32
H7A	6894	4501	766	43
H7B	5534	3293	637	43
H8A	8661	5726	1313	48
H8B	10002	5570	1666	48
H8C	8102	6302	1726	48
H10	6024	5683	2247	31
H12	9784	2948	2292	36
H13A	9274	3464	2950	38
H13B	7915	2210	2850	38
H15	4965	3009	2560	31
H16A	2458	4268	2664	41
H16B	3509	5745	2723	41
H17A	2759	3734	3284	38
H17B	3608	5283	3350	38

Atom	*x*	*y*	*z*	U(eq)
H18	5411	2646	3255	33
H19A	8137	5763	3170	55
H19B	6283	6326	3004	55
H19C	7901	6003	2720	55
H20A	8065	2369	3712	80
H20B	8499	3622	4006	80
H20C	8998	3797	3564	80
H21	6426	5116	3684	40
H22A	3755	3833	3892	44
H22B	5039	2548	4006	44
H23A	6388	3882	4482	42
H23B	5465	5316	4335	42
H24A	3601	2980	4670	41
H24B	2641	4372	4511	41
H26A	6537	4333	5131	63
H26B	5288	3064	5275	63
H26C	5412	4506	5516	63
H27A	2186	4988	5494	65
H27B	1925	3480	5290	65
H27C	1141	4889	5097	65

Reference

1. O.V. Dolomanov and L.J. Bourhis and R.J. Gildea and J.A.K. Howard and H. Puschmann, Olex2: A complete structure solution, refinement and analysis program, *J. Appl. Cryst.*, (2009), **42**, 339-341.
